# Functional Difference between Sustained and Transient Modulations of Cognitive Control in the Simon Task: Evidence from False Alarm Responses on No-Go Trials

**DOI:** 10.1371/journal.pone.0081804

**Published:** 2013-11-26

**Authors:** Kunihiro Hasegawa, Shin’ya Takahashi

**Affiliations:** Department of Psychology, Nagoya University, Nagoya, Japan; The Ohio State University, Center for Cognitive and Brain Sciences, Center for Cognitive and Behavioral Brain Imaging, United States of America

## Abstract

Cognitive control in response compatibility tasks is modulated by the task context. Two types of contextual modulations have been demonstrated; sustained (block-wise) and transient (trial-by-trial). Recent research suggests that these modulations have different underlying mechanisms. This study presents new evidence supporting this claim by comparing false alarm (FA) responses on no-go trials of the Simon task between the sustained and transient contexts. In Experiment 1, the sustained context was manipulated so that a block included a larger number of incongruent trials. Results showed that participants made more FA responses by the hand opposite to the stimulus location. This suggests a generation of response bias in which the task-irrelevant location information is utilized in a reversed manner (i.e., to respond with the right hand to a stimulus presented on the left side and vice versa). Next, Experiment 2 examined the effect of the transient context and found that overall FA rate was lower when a no-go trial was preceded by an incongruent trial than by a congruent trial, whereas such response bias as that shown in Experiment 1 was not demonstrated. This suggests that the transient conflict context enhances inhibition of the task-irrelevant process but does not make the task-irrelevant information actively usable. Based on these results, we propound two types of cognitive control modulations as adaptive behaviors: response biasing based on utilization of the task-irrelevant information under the sustained conflict context and transient enhancement of inhibition of the task-irrelevant process based on the online conflict monitoring.

## Introduction

Cognitive control plays a critical role in our goal-directed behavior through conflict detection, resolution, and adaptation. In a laboratory situation, cognitive control is often examined using cognitive conflict tasks with a congruency effect as an index. For example, in the Simon task [1], reaction time (RT) on incongruent trials (e.g., participants respond with their right hand to a color stimulus presented on the left side) is likely to be longer than RT on congruent trials (e.g., participants respond with their right hand to the stimulus presented on the right side). This effect is considered to result from an automatic activation for responding with the hand compatible with the spatial location of the target stimulus. In the case where the side of a required response and the target location are incongruent, a conflict arises that is only resolved when the automatically-triggered location-based response is overcome, thereby resulting in longer RT [2]. Thus, the cognitive conflict plays a major role in the congruency effect (difference between RTs for the congruent and incongruent trials), indicating the cost of cognitive control.

The important aspect of cognitive control as an adaptive behavior is that it is modulated by the task context. It is recognized that the congruency effect decreases when a congruent/incongruent trial is immediately preceded by an incongruent trial relative to when preceded by a congruent trial [3]. This is called the *conflict adaptation*, or the *Gratton effect*. This transient modulation of cognitive control is regarded as an adaptation to the trial-by-trial context. As an account of the transient modulation, the conflict monitoring theory [4,5] has been widely accepted by researchers because it collectively explains behavioral evidences, neural bases, and computing simulation. This theory assumes the trigger system of the control modulation and reveals the neural basis of this detection-modulation system. According to this, the control modulation is triggered by conflict detection in the anterior cingulate cortex, which recruits the control demand. This demand is then used in the prefrontal cortex to adjust processing of information that is relevant or irrelevant to the required task; the task-relevant process is enhanced [6,7] and the task-irrelevant process is inhibited [8,9]. Consequently, the congruency effect is weakened after an incongruent trial. 

In addition to the effect by transient (trial-by-trial) context, sustained (block-wise) context is also known to influence the congruency effect. This is called the *conflict context effect* or the *proportion congruency effect*. The congruency effect tends to be smaller in an experimental block containing a larger number of incongruent trials than in a block with a larger number of congruent trials [3]. Furthermore, in such an experimental design with extremely large proportion of incongruent trials, the congruency effect has been shown not only to disappear but also to reverse [10-12]. In this case, known as the reverse congruency effect, RT on the congruent trials becomes longer than RT on the incongruent trials.

A critical, but unsettled, question is whether the sustained and transient modulations are caused by the same underlying mechanism. According to the conflict monitoring theory, the transient modulation is achieved by conflict monitoring on a trial-by-trial basis as described above, and the sustained modulation is regarded as an accumulation of such a transient effect. However, recent studies emphasize that these two types of contextual modulations have different mechanisms [10,13-16]. According to these dual control accounts, the control modulation for transient trial-by-trial sequence is explained as an enhancement of inhibition of the task-irrelevant process (micro-adjustment [16]; reactive-control [13,14]. On the other hand, the control modulation for sustained conflict context is explained as boosting of the task-relevant process (macro-adjustment [16]; proactive-control [13,14]). However, our resent study [10] showed that control modulation for the sustained conflict context, as well as for the transient context, was mediated by the task-irrelevant process. Consequently, the present study focuses on the difference between the effects of sustained conflict context and transient trial-by-trial sequence on the task-irrelevant process. 

The important behavioral evidence that suggests different mechanisms between the sustained and transient modulations is the aforementioned reverse congruency effect. The reverse congruency effect generated by the sustained context cannot be explained by any idea based on accumulation of the effect generated by the transient context [4,5] or boosting of the task-relevant process (macro-adjustment/proactive-control) [13,14,16]. This is because, regardless of how much the task-relevant process was enhanced, the congruency effect would not reverse. Similarly, if the task-irrelevant process was completely inhibited, then the response should be activated only by the task-relevant feature in both the congruent and incongruent trials, which means that these trial types no longer have different features (i.e., task-irrelevant information) to process; this would result in no congruency effect, not the reverse congruency effect. 

To explain the reverse congruency effect, we need an idea that regards the sustained effect as more than simple accumulation of the transient effect. Contingency learning account, which was originally advocated for the Stroop task [17,18], may be one convincing explanation for the Simon task as well. This account explains the sustained conflict context effect in terms of the utilization bias of the task-irrelevant information based on the contingency learning of association between the task-irrelevant information and the response mapping. For example, in the Stroop task, if the word “RED” is presented most frequently in orange, an incongruent response bias (i.e., the word “RED” is predictive of orange response) may be generated. In this case, any form of “active utilization,” not “passive invalidation,” of the task-irrelevant information must be considered.

Therefore, in this study, we focused on processing of the task-irrelevant information during the sustained modulation, which has not been extensively discussed so far. Specifically, we aimed to obtain evidences that indicate the active utilization of the task-irrelevant information when the reverse congruency effect was observed. For instance, in the Simon task, under the condition in which an experimental block contains extremely large proportion of incongruent trials, a potential reactive bias toward responding with the hand opposite to the stimulus location may be generated as a result of experiencing a number of incongruent trials. This in turn may result in the reverse congruency effect, since such a response bias is more advantageous to the incongruent than to the congruent trials.

To demonstrate the response bias that may reflect active utilization of the task-irrelevant information, we examined false alarm (FA) responses on no-go trials within the Simon task. Participants are instructed to respond to a red or green stimulus by pressing corresponding keys, while ignoring the presentation side of the stimulus that may agree or disagree with the side of the key to be pressed (i.e., the hand to be used). In addition to these trials comprising the typical Simon task, no-go trials were included, on which a gray stimulus is presented on the left or right side of a screen, and participants are not required to press any keys. Thus, the no-go stimulus contains information about the stimulus location but not about stimulus color that defines the hand to be used. If the response bias to the side opposite to the stimulus location was involved in generation of the sustained modulation, then the biased response, which emerges as an FA on no-go trials, should probably be elicited from the hand opposite to the stimulus side when the block contains a larger number of incongruent trials. Thus, we examined the type of FA responses (same and opposite) on no-go trials as well as overall FA rates and the RTs on normal Simon trials (congruent and incongruent trials) in two experiments in which the sustained and transient contexts were manipulated.

In addition, the repetition priming effect [19,20] should be considered here. It states that RT on the partial repetition trials (in a condition where repetition applies from the preceding trial either to a task-relevant stimulus feature corresponding to a required response or to the stimulus location) is likely to be longer than RT on the complete change trials (both the task-relevant stimulus feature and the stimulus location are changed) and complete repetition trials (both the task-relevant stimulus feature and the stimulus location are repeated). We would be able to assume that this effect is not critical for the no-go trials highlighted in the present study, because no-go stimulus has no task-relevant feature and thus the repetition from the preceding trial can be possible only for its location. Nevertheless, for more elaborated discussion, we conducted additional analysis using data of the complete change no-go trials only, and compared them with those of all (complete change and partial repetition) no-go trials. 

### Ethics Statement

This research has approved by the review board of the Department of Psychology, Graduate School of Environmental Studies, Nagoya University. All participants provided written informed consent. They gave permission to use their data in the analysis. 

## Experiment 1

In Experiment 1, each participant performed the Simon task over two blocks of trials; one 80%-congruent and the other 80%-incongruent. The 80%-congruent block contained 80% congruent, 10% incongruent, and 10% no-go trials. The 80%-incongruent block contained 80% incongruent, 10% congruent, and 10% no-go trials. In addition to the RT on congruent and incongruent trials, FA rate in each FA type (same or opposite) on no-go trials was examined as an index of the response bias.

### Methods

#### Participants

Eighteen undergraduate and graduate students at Nagoya University were recruited as participants (10 women and 8 men; 21–32 years of age, *M* = 24.1 years). All participants reported normal or corrected-to-normal vision. 

#### Apparatus and Stimuli

The stimuli were displayed on a CRT monitor (GDM-F520, Sony) controlled by a computer (MB324J/A, Apple). Participants viewed the stimulus with their face on a chin-rest. The viewing distance was 95.5 cm. 

We wrote our experiments in Matlab (Mathworks), using the Psychophysics Toolbox extensions [21-23]. A disk (1° of visual angle) was displayed in one of three colors (red, green, and gray) on the left or right side (2° away from the center) of the monitor. The background color was consistently black.

Data was analyzed by using R (http://www.r-project.org/).

#### Design and Procedure

Participants were asked to press the “C” key on a keyboard with the left index finger when the stimulus color was green, the “M” key with the right index finger when the stimulus color was red, and to refrain from pressing any key when the stimulus color was gray. This stimulus-response mapping was the same for all participants. The stimulus was presented until the response was offered or 1000 ms passed without a response. A response-stimulus interval (RSI) was 800 ms, during which a white fixation cross was presented at the center of the monitor.

Participants performed two separate blocks (80%-congruent and 80%-incongruent blocks), each consisting of 480 experimental trials. The 80%-congruent block had 384 congruent trials (a green disk was presented on the left side or a red disk on the right side), 48 incongruent trials (a green disk was presented on the right side or a red disk on the left side), and 48 no-go trials (a gray disk was presented either on the left or right side). The 80%-incongruent block had 48 congruent trials, 384 incongruent trials, and 48 no-go trials. The block order was counterbalanced and the trial order in each block was randomized among participants. Participants were permitted a short rest between blocks. They were not informed about the proportion of congruent/incongruent trials in a block. In fact, none of the participants noticed this manipulation until the debriefing after the experiment.

### Results

#### Reaction Time on Congruent and Incongruent Trials

**Figure 1:**
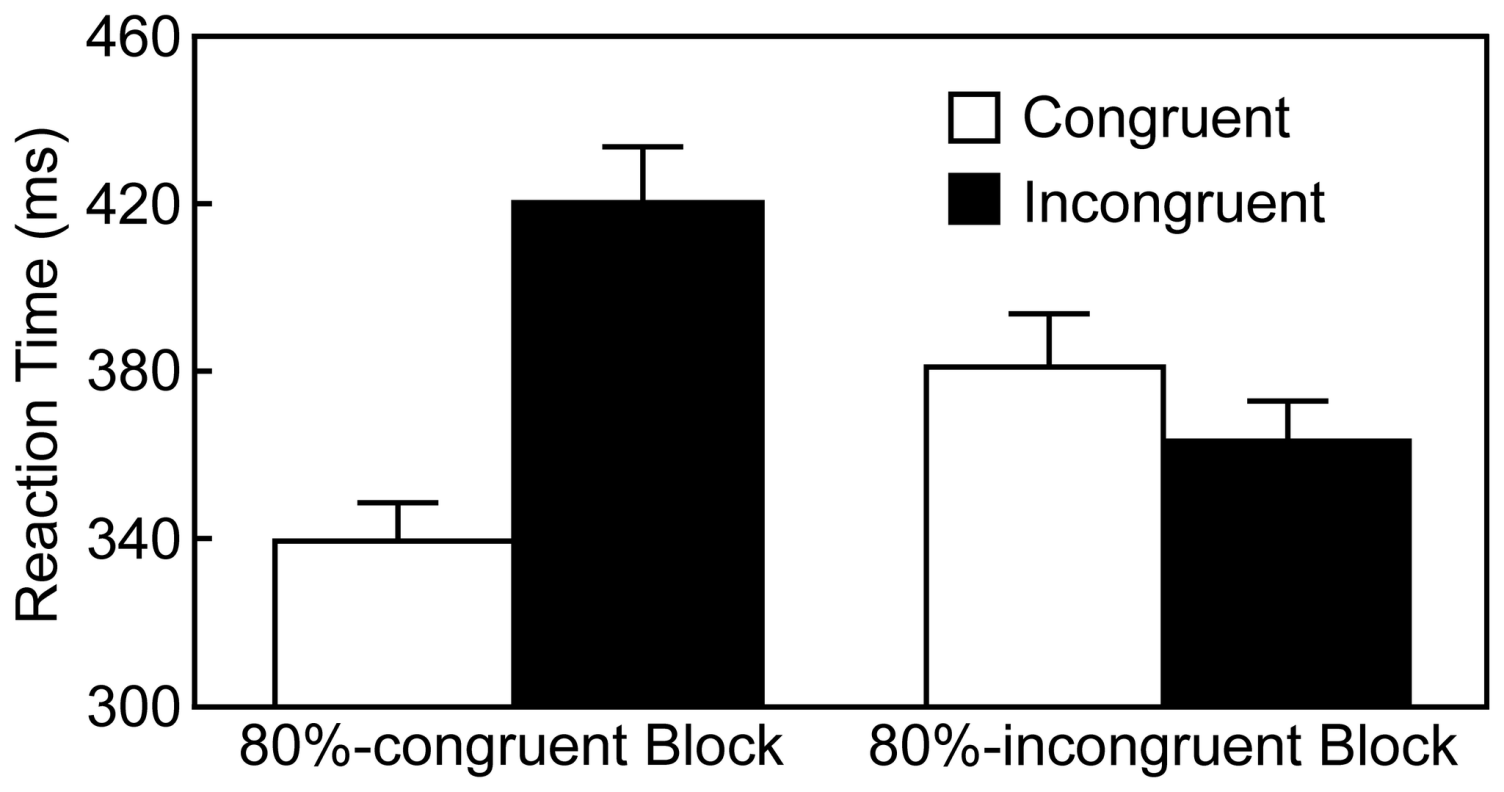
Mean reaction times in Experiment 1; error bars indicate standard errors of the mean.

Trials invoked longer or shorter RT than the mean RT ± 2.5 *SD* for each participant (counted 1.3% of congruent and incongruent trials when averaged across participants) were excluded from the analyses. In addition, data were also discarded when an incorrect response was made on the current trial or on the immediately preceding trial (counted 6.7% across participants; see below for error rates of the current trial in each experimental condition). The RTs were analyzed by a two-way repeated measures analysis of variance (ANOVA) with the block type (80%-congruent and 80%-incongruent) and the current trial type (congruent and incongruent). Figure 1 illustrates the results. There was a significant main effect of the current trial type [*F* (1, 17) = 28.47, *p* < .001, *η* ^2^ = .08] and a significant interaction between two factors [*F* (1, 17) = 87.26, *p* < .001, *η* ^2^ = .20]. The main effect of the block type was not significant [*F* (1, 17) = 2.54, *p* = .12, *η* ^2^ = .01]. Post-hoc analyses using Ryan's method [24] indicated that, when the block type was 80%-congruent, RT on the incongruent trials was longer than RT on the congruent trials (*p* < .001, Cohen's d = 1.71), and vice versa when the block type was 80%-incongruent (*p* = .02, Cohen's d = 0.40). In addition, RT on the congruent trials was shorter in the 80%-congruent than in the 80%-incongruent block (*p* < .001, Cohen's d = 0.92), and vice versa on the incongruent trials (*p* < .001, Cohen's d = 1.16). Therefore, we confirmed the sustained modulation of cognitive control, because the reverse congruency effect was clearly observed in the 80%-incongruent block.

#### Error Rates on Congruent and Incongruent Trials

Mean error rates were analyzed by a repeated measures ANOVA with the block type (80%-congruent and 80%-incongruent) and the current trial type (congruent and incongruent). There was a significant main effect of the block type [*F* (1, 17) = 9.65, *p* = .006, *η* ^2^ = .01], a significant main effect of the current trial type [*F* (1, 17) = 6.39, *p* = .02, *η* ^2^ = .02], and a significant interaction between two factors [*F* (1, 17) = 6.08, *p* = .03, *η* ^2^ = .08]. Post-hoc analyses using Ryan's method [24] indicated that, when the block type was 80%-congruent, error rate on the incongruent trials was higher than that on the congruent trials (6.6% vs. 1.0%, *p* = .002, Cohen's d = 0.79). However when the block type was 80%-incongruent, there was no significant difference between error rates on the congruent and incongruent trials (3.1% vs. 1.4%, *p* = .31, Cohen's d = 0.34). In addition, error rate on the incongruent trials was lower in the 80%-incongruent block than in the 80%-congruent block (1.4% vs. 6.6%, *p* = .002, Cohen's d = 0.73). However error rates on the congruent trials did not differ between the 80%-congruent and 80%-incongruent blocks (1.0% vs. 3.1%, *p* = .18, Cohen's d = 0.43). 

#### False Alarm on No-go Trials

**Figure 2:**
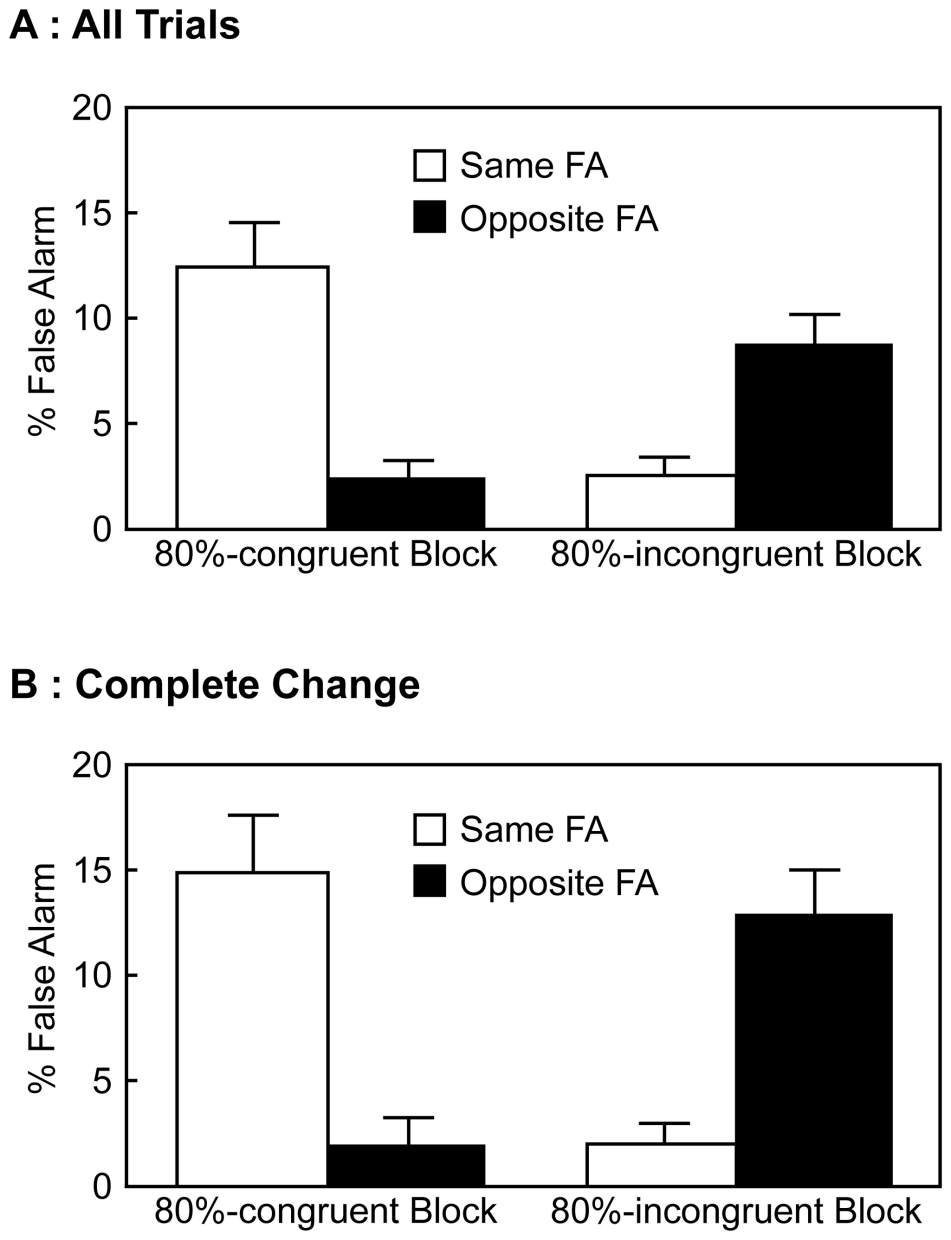
Mean FA rates on no-go trials in Experiment 1; error bars indicate standard errors of the mean. A: FA rates on all no-go trials. B: FA rates on the complete change no-go trials.

The mean FA rates on no-go trials are depicted in Figure 2A. The FA rates were analyzed by a repeated measures ANOVA with the block type (80%-congruent and 80%-incongruent) and the FA type (same and opposite). The “same FA” refers to an FA response made by the hand on the same side as the stimulus location and the “opposite FA” is that made by the hand opposite to the stimulus location. There was a significant main effect of the block type [*F* (1, 17) = 8.32, *p* = .01, *η* ^2^ = .02], a significant main effect of the FA type [*F* (1, 17) = 7.01, *p* = .02, *η* ^2^ = .02], and a significant interaction between two factors [*F* (1, 17) = 40.22, *p* < .001, *η* ^2^ = .31]. Post-hoc analyses using Ryan's method [24] indicated that, when the block type was 80%-congruent, the same FA was more frequent than the opposite FA (*p* < .001, Cohen's d = 1.57). In contrast, when the block type was 80%-incongruent, the opposite FA was more frequent than the same FA (*p* < .001, Cohen's d = 1.18). In addition, the same FA rate was higher in the 80%-congruent than 80%-incongruent block (*p* < .001, Cohen's d = 1.56). Conversely, the opposite FA rate was lower in the 80%-congruent than 80%-incongruent block (*p* < .001, Cohen's d = 1.19). 

Furthermore, data of only the complete change no-go trials were analyzed. These are no-go trials whose stimulus location is different from that of the preceding trial. Results are shown in Figure 2B, and were analyzed by the same ANOVA as before. There was a significant interaction between two factors [*F* (1, 17) = 33.782, *p* < .001, *η* ^2^ = .36]. The main effect of the block type and the main effect of the FA type were not significant [*F* (1, 17) = 0.53, *p* = .48, *η* ^2^ < .01; *F* (1, 17) = 0.44, *p* = .52, *η* ^2^ < .01]. Post-hoc analyses using Ryan's method [24] indicated that, when the block type was 80%-congruent, the same FA was more frequent than the opposite FA (*p* < .001, Cohen's d = 1.46). In contrast, when the block type was 80%-incongruent, the opposite FA was more frequent than the same FA (*p* < .001, Cohen's d = 1.47). In addition, the same FA rate was higher in the 80%-congruent than 80%-incongruent block (*p* < .001, Cohen's d = 1.48). Conversely, the opposite FA rate was lower in the 80%-congruent than 80%-incongruent block (*p* < .001, Cohen's d = 1.44).

#### Correlation between the response biasing and the reverse congruency effect

**Figure 3:**
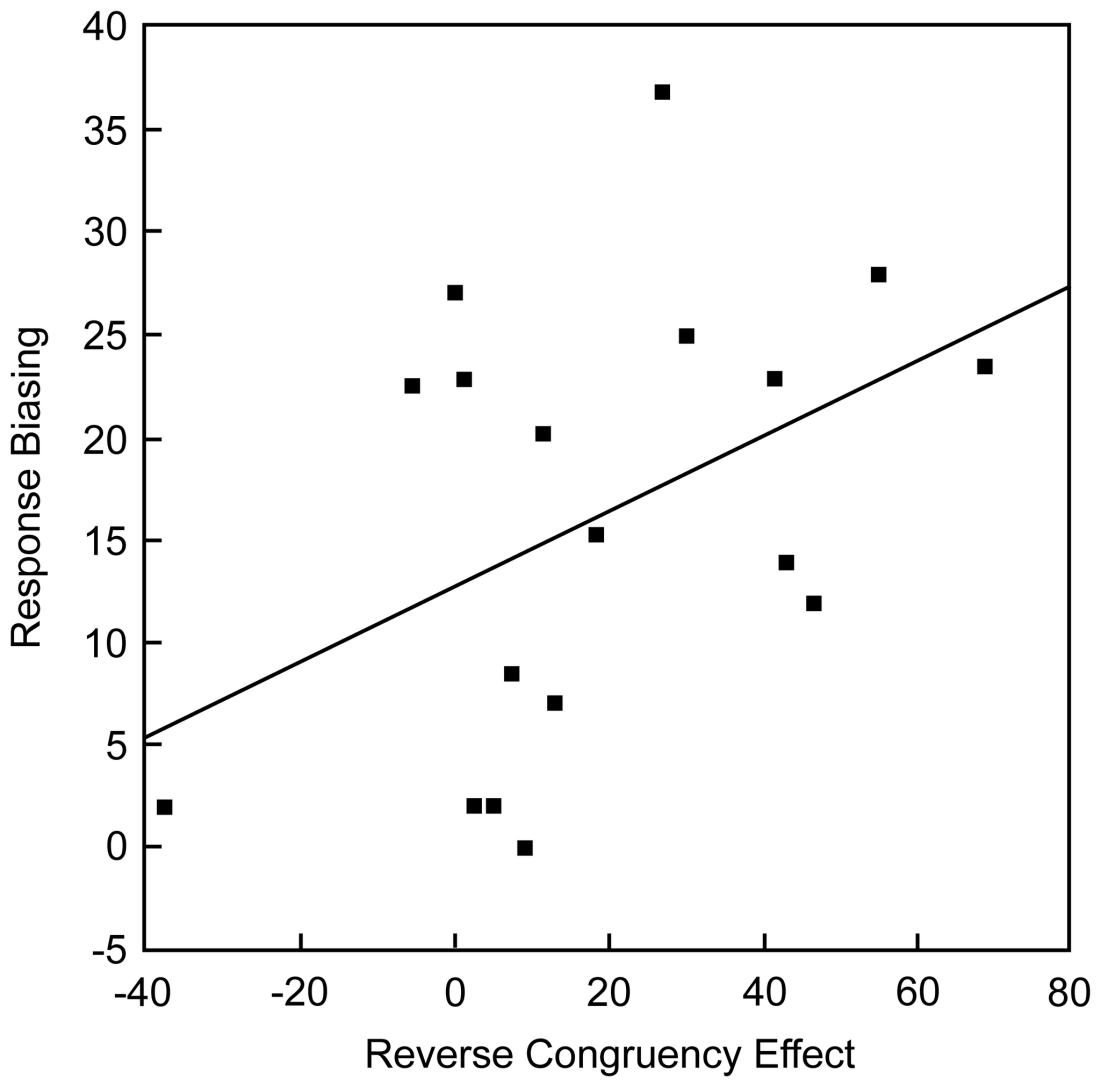
Correlation between the response biasing and the reverse congruency effect in the 80%-incongruent block of Experiment 1.

If the reverse congruency effect was caused by the response biasing, it is assumed that the individual having greater response biasing would yield greater reverse congruency effect. To test this assumption, the degree of contralateral biasing (opposite FA rate minus same FA rate) and the degree of reverse congruency effect (RT on congruent trials minus RT on incongruent trials) in the 80%-incongruent block was obtained for each participant, and the correlation of these values was calculated. As shown in Figure 3, this correlation was significant (Pearson's *r* = .56, *p* = .02).

### Discussion

In Experiment 1, effect of the sustained modulation of cognitive control was clearly observed; RT was shorter on the congruent than incongruent trials in the 80%-congruent block, whereas this trend was reversed in the 80%-incongruent block. And, more importantly, the frequency of FA response on the same side as the stimulus location was higher than that on the opposite side in the 80%-congruent block, and vice versa in the 80%-incongruent block. This result suggests a generation of the utilization bias of task-irrelevant information in the 80%-incongruent block [16-18]. This tendency of reversing FA rates was found regardless of whether the analysis was done for data of all no-go trials or for data of only the complete change no-go trials, suggesting that the effect of the repetition priming was small. In addition, there was a significant positive correlation between the response biasing and the reverse congruency effect. These results fit with the response biasing account based on the contingency learning of association between the task-irrelevant information and the response mapping [17,18]. 

Before concluding that the response bias is a cause of the sustained modulation, possibility of its being generated by the trial-by-trial adaptation, not by the sustained conflict context, should be eliminated. Otherwise, the effect of block type observed in this experiment might be confounded with the effect of transient (trial-by-trial) modulation, since not only the proportion of congruent and incongruent trials but also the probable relationship of congruency between successive two trials would have been varied by the block type. Consequently, in Experiment 2, we examine the effect of trial-by-trial modulation of cognitive control on the same response bias as that shown in Experiment 1.

## Experiment 2

In Experiment 2, to examine the effect of only the transient modulation (i.e., to exclude the effect of the sustained modulation), the same proportion of congruent and incongruent trials were presented: 45% congruent, 45% incongruent, and 10% no-go trials. FA rates on no-go trials were examined separately for the preceding trial types, congruent and incongruent. If the response bias observed in Experiment 1 could be generated by the trial-by-trial, not by the sustained, modulation, similar bias should be observed even when the congruent and incongruent trials were presented with equal probability. Furthermore, according to the dual control account [13,14,16], inhibition of the task-irrelevant processing would be enhanced by the transient conflict context. As such, if there was a transient enhancement of inhibition, the overall FA rate on no-go trials should be lower when preceded by an incongruent trial than by a congruent trial.

### Methods

#### Participants

Eighteen undergraduate and graduate students at Nagoya University were recruited as participants (10 women and 8 men; 21–32 years of age, *M* = 24.3 years). All participants reported normal or corrected-to-normal vision. 

#### Apparatus, Stimuli, Design, and Procedure

Apparatus, the stimuli, experimental design, and the procedure were the same as in Experiment 1 with the following exception. Participants performed 480 experimental trials in one block: 216 congruent, 216 incongruent, and 48 no-go trials. The trial order was pseudo-randomized for each participant to present equal number of no-go trials after the congruent and incongruent trials (i.e., 24 trials each). 

### Results

#### Reaction Time on Congruent and Incongruent Trials

**Figure 4:**
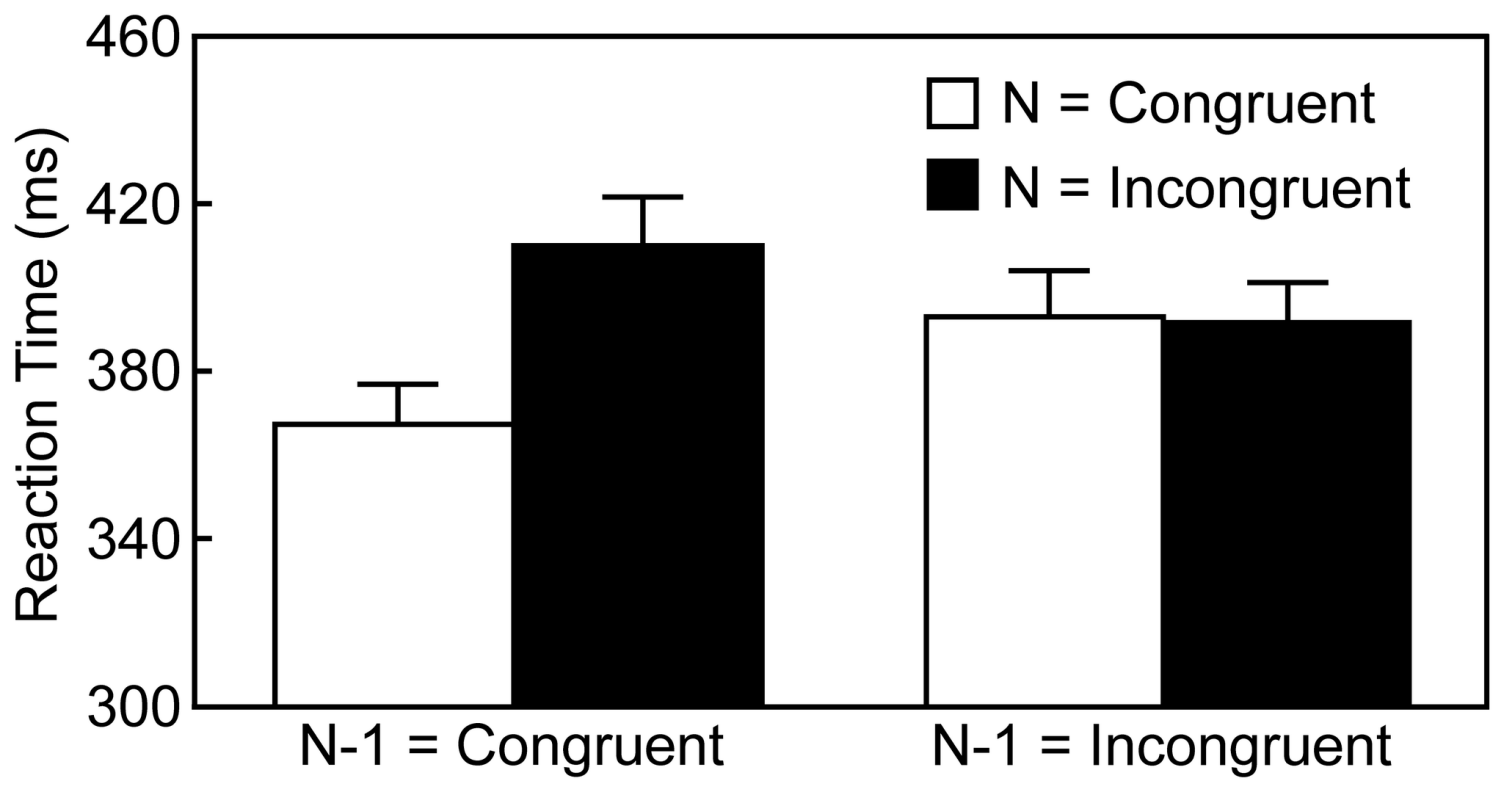
Mean reaction times in Experiment 2; error bars indicate standard errors of the mean.

Trials invoked longer or shorter RT than the mean RT ± 2.5 *SD* for each participant (counted 1.3% of congruent and incongruent trials when averaged across participants) were excluded from the analyses. In addition, data were also discarded when an incorrect response was made on the current trial or on the immediately preceding trial (counted 9.3% across participants; see below for error rates of the current trial in each experimental condition). The RTs were analyzed by a two-way repeated measures ANOVA with the preceding trial type (congruent and incongruent) and the current trial type (congruent and incongruent). Figure 4 shows the results. There was a significant main effect of the current trial type [*F* (1, 17) = 56.81, *p* < .001, *η* ^2^ = .05] and a significant interaction between two factors [*F* (1, 17) = 48.09, *p* < .001, *η* ^2^ = .07]. The main effect of the preceding trial type was not significant [*F* (1, 17) = 2.26, *p* = .15, *η* ^2^ = .00]. Post-hoc analyses using Ryan's method [24] showed that, when the preceding trial type was congruent, RT on the incongruent current trials was longer than RT on the congruent current trials (*p* < .001, Cohen's d = 1.04). In contrast, when the preceding trial type was incongruent, RTs on the congruent and incongruent current trials did not differ significantly (*p* = .54, Cohen's d = 0.06). In addition, RT on the congruent current trials was shorter when the preceding trial type was congruent than incongruent (*p* < .001, Cohen's d = 0.64), and vice versa on the incongruent current trials (*p* < .001, Cohen's d = 0.45). Therefore, we confirmed the transient modulation of cognitive control in this experiment, because the congruency effect was observed only when the preceding trial type was congruent.

#### Error Rates on Congruent and Incongruent Trials

Mean error rates were analyzed by a repeated measures ANOVA with the preceding trial type (congruent and incongruent) and the current trial type (congruent and incongruent). The main effect of the preceding trial type [*F* (1, 17) = 1.94, *p* = .18, *η* ^2^ = .01] and the main effect of the current trial type [*F* (1, 17) = 2.85, *p* = .11, *η* ^2^ = .04] were not significant. The interaction between two factors was significant [*F* (1, 17) = 28.18, *p* < .001, *η* ^2^ = .17]. Post-hoc analyses using Ryan's method [24] indicated that, when the preceding trial type was congruent, error rate on the incongruent current trials was higher than that on the congruent current trials (8.2% vs. 1.8%, *p* < .001, Cohen's d = 1.15). In contrast, when the preceding trial type was incongruent, there was no significant difference between error rates on the congruent and incongruent current trials (5.1% vs. 2.8%, *p* = .12, Cohen's d = 0.60). In addition, error rate on the congruent current trials was lower when the preceding trial type was congruent than incongruent (1.8% vs. 5.1%, *p* = .006, Cohen's d = 0.90), and vice versa on the incongruent current trials (8.2% vs. 2.8%, *p* < .001, Cohen's d = 0.95).

#### False Alarm on No-go Trials

**Figure 5:**
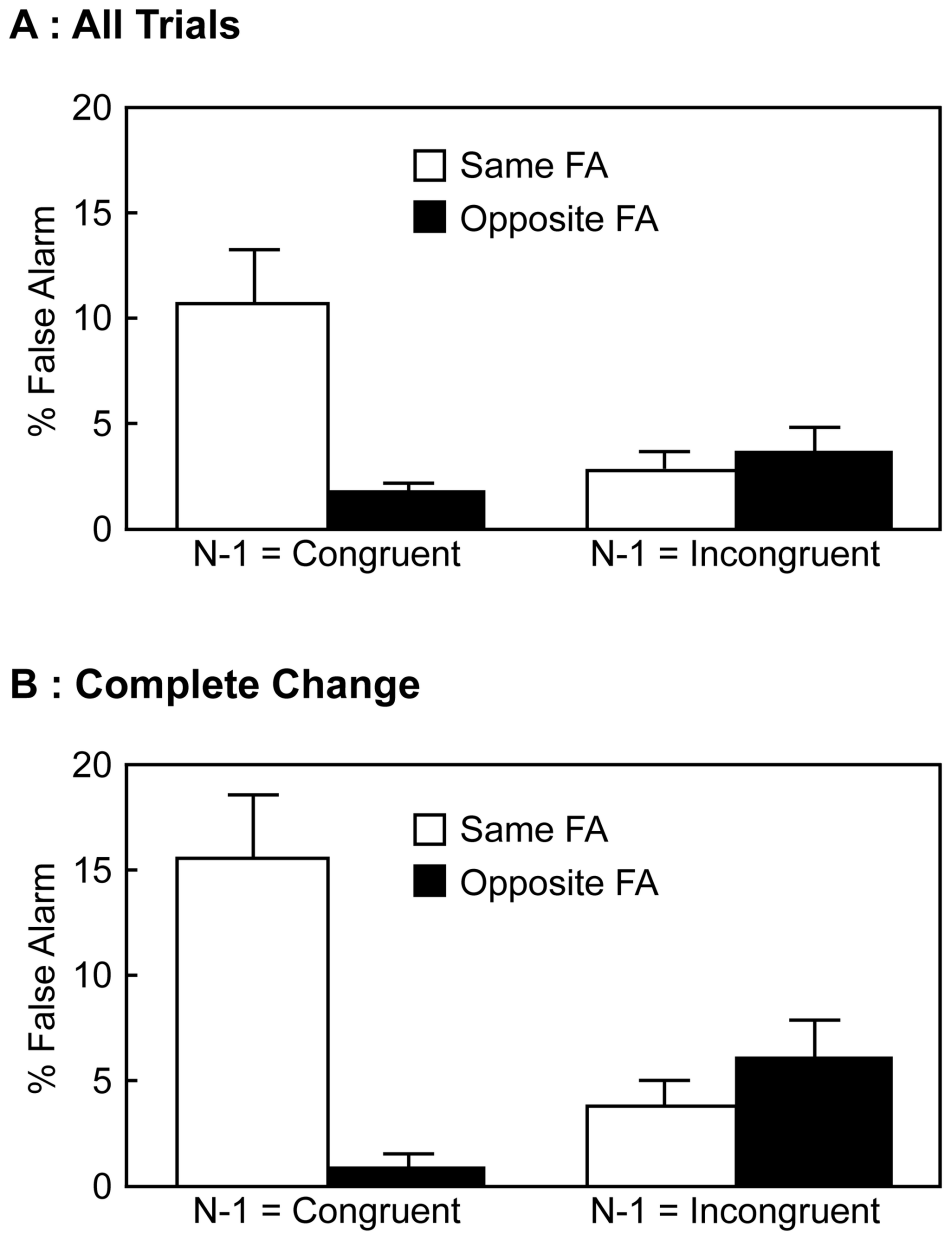
Mean FA rates on no-go trials in Experiment 2; error bars indicate standard errors of the mean. A: FA rates on all no-go trials. B: FA rates on the complete change no-go trials.

The mean FA rates on the no-go trials are shown in Figure 5A. The FA rates were analyzed by a repeated measures ANOVA with the preceding trial type (congruent and incongruent) and the FA type (same and opposite). There was a significant main effect of the preceding trial type [*F* (1, 17) = 4.89, *p* = .04, *η* ^2^ = .04], a significant main effect of the FA type [*F* (1, 17) = 6.23, *p* = .02, *η* ^2^ = .08], and a significant interaction between two factors [*F* (1, 17) = 14.143, *p* = .002, *η* ^2^ = .12]. Post-hoc analyses using Ryan's method [24] showed that, when the preceding trial type was congruent, the same FA was more frequent than the opposite FA (*p* < .001, Cohen's d = 1.17). In contrast, when the preceding trial type was incongruent, there was no difference between the rates of two FA types (*p* = .64, Cohen's d = 0.21). In addition, the same FA rate was higher after congruent than incongruent trials (*p* < .001, Cohen's d = 0.97), whereas the opposite FA rate did not differ significantly between the preceding trial types (*p* = .30, Cohen's d = 0.57). 

Next, as in Experiment 1, mean FA rates on only the complete change no-go trials were calculated (Figure 5B), and analyzed by a repeated measures ANOVA with the preceding trial type and the FA type. There was a marginally significant main effect of the preceding trial type [*F* (1, 17) = 3.45, *p* = .08, *η* ^2^ = .03], a significant main effect of the FA type [*F* (1, 17) = 15.00, *p* = .001, *η* ^2^ = .10], and a significant interaction between two factors [*F* (1, 17) = 24.359, *p* < .001, *η* ^2^ = .20]. Post-hoc analyses using Ryan's method [24] showed that, when the preceding trial type was congruent, the same FA was more frequent than the opposite FA (*p* < .001, Cohen's d = 1.53). In contrast, when the preceding trial type was incongruent, there was no difference between the rates of two FA types (*p* = .34, Cohen's d = 0.37). In addition, the same FA rate was higher after congruent than incongruent trials (*p* < .001, Cohen's d = 1.16), and the opposite FA rate was lower after congruent than incongruent trials (*p* = .05, Cohen's d = 0.99).

#### Correlation between the response biasing and the reverse congruency effect

**Figure 6:**
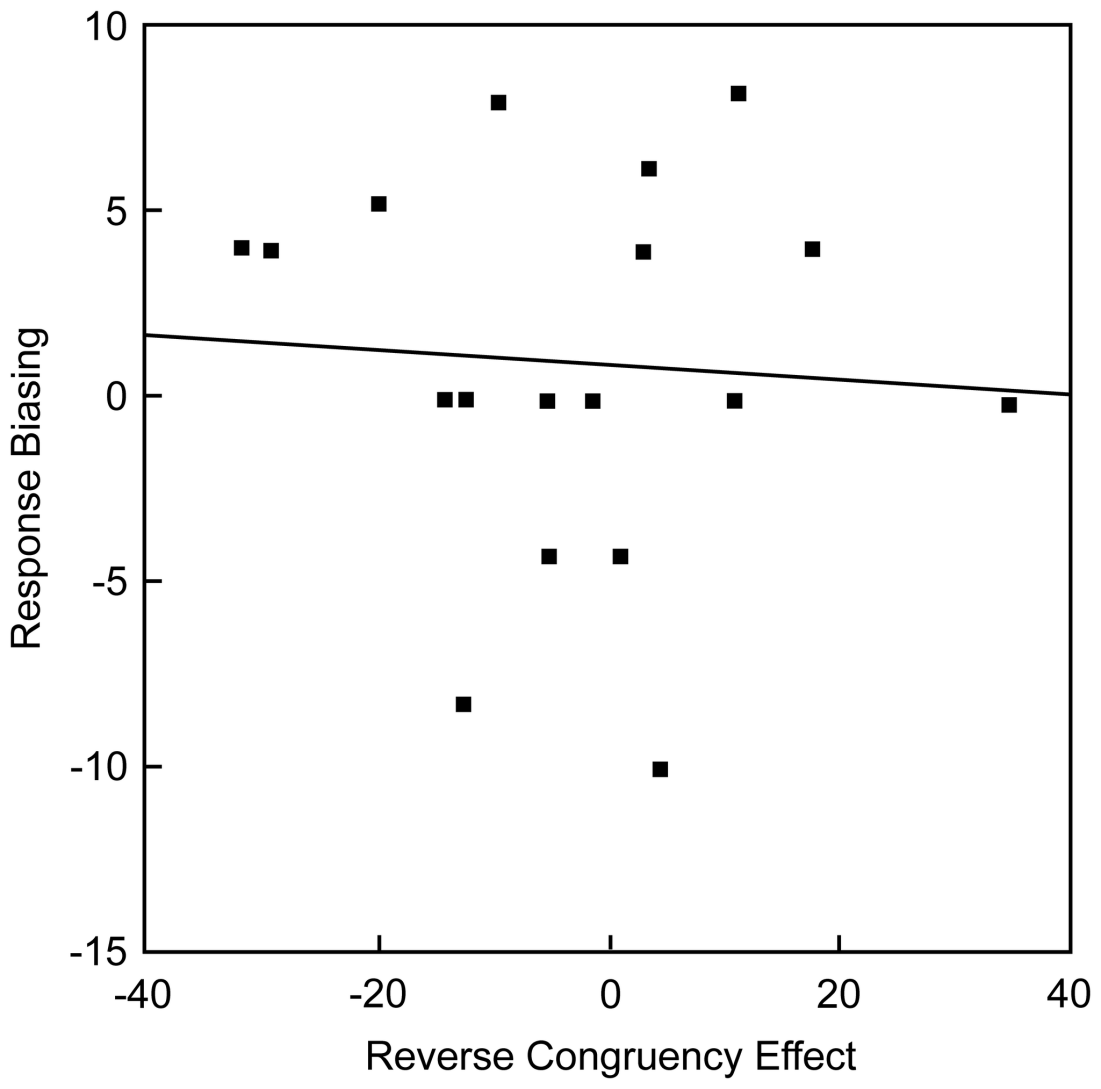
Correlation between the response biasing and the reverse congruency effect when the preceding trial type was incongruent in Experiment 2.

Though significant reverse congruency effect was not found when the preceding trial type was incongruent, the same correlational analysis as that in the 80%-incongruent block of Experiment 1 was conducted between the degree of contralateral biasing and the degree of the reverse congruency effect on trials preceded by an incongruent trial. As shown in Figure 6, this correlation was not significant (Peason's *r* = -.07, *p* = .78).

### Discussion

The present results indicated that RT on congruent trials was shorter than RT on incongruent trials when the preceding trial type was congruent, whereas no congruency effect was observed when the preceding trial type was incongruent; thus, the transient modulation of cognitive control was shown. More importantly, the FA analysis demonstrated that, unlike Experiment 1, the preceding trial type did not reverse the dominant FA type. When the preceding trial type was congruent, FAs were made more frequently by the hand on the same side as the stimulus location than by the hand on the opposite side, which was the same result as in the 80%-congruent block of Experiment 1. Conversely, when the preceding trial type was incongruent, the same and opposite FA rates did not differ significantly, although the former was slightly lower than the latter. Furthermore, the overall FA rate was significantly lower after the incongruent trial than after the congruent trial, supporting the dual control account [13,14,16] that assumes response inhibition for the task-irrelevant information is enhanced by the transient context. These results are decisively different from those in the 80%-incongruent block of Experiment 1, in which the opposite FA rate was significantly higher than the same FA rate, and fit with inhibition account [8] and the micro-adjustment/reactive-control supposed by the dual control account [13,14,16]. 

Collectively, these results suggest that location information of the no-go stimulus would automatically activate the same side response to induce the same FA when it was immediately preceded by the congruent trial, whereas such an automatic activation was inhibited after the incongruent trial. Consequently, the present results indicate that the response bias observed in Experiment 1 was primarily due to the sustained, rather than the transient, modulation of cognitive control. The transient conflict context would trigger inhibition of the task-irrelevant processing, but wouldn’t render the task-irrelevant information actively usable to lead to more opposite FAs and generate the reverse congruency effect as in the case of the sustained conflict context.

## General Discussion

The present study investigated the difference in processing of the task-irrelevant information of the Simon task between sustained and transient contexts by analyzing FA responses on no-go trials. Experiment 1 demonstrated that the rate of FA response for the same side as the stimulus location was higher than that for the opposite side in the 80%-congruent block, and vice versa in the 80%-incongruent block, suggesting that response bias for the side opposite to the stimulus location was generated by experiencing a number of incongruent trials. However, such a response bias was not found in Experiment 2, which examined the trial-by-trial context. When the transient context was congruent (no-go trial was immediately preceded by a congruent trial), the same FA rate was higher than the opposite FA rate. However, when the transient context was incongruent, the same and opposite FA rates did not differ, contrasting with the sustained incongruent context (80%-incongruent block of Experiment 1) in which the opposite FA rate exceeded the same FA rate.

Together, the present results show that response bias was dependent on the overall proportion of congruent and incongruent trials. In other words, this bias may be a causal factor of the reverse congruency effect by the sustained modulation of cognitive control observed in Experiment 1. To support this view, a significant correlation was found between the degree of contralateral biasing and the degree of reverse congruency effect in the 80%-incongruent block. These results are consistent with the utilization bias account based on the contingency learning [17,18]. Such a contingency learning seems to depend on property of the overall context formed by a number of prior trials rather than the immediate influence from the preceding trial, since the effect of the repetition priming was found to be small. In addition, in Experiment 1, no instruction about the proportion of congruent and incongruent trials was provided to the participants. In fact, subjective reports obtained following the experiment revealed that all participants were unaware of the variation in the proportion of congruent/incongruent trials between blocks, suggesting that the response bias was implicitly and automatically generated.

In Experiment 2, FA rate on no-go trials was lower after the incongruent trial than after the congruent trial. This result is consistent with the inhibition account [8] or the micro/reactive control account [13,14,16], which assumes that the demand for control generated by experiencing conflict in the preceding trial (i.e., short-term conflict context) inhibits response activation by the task-irrelevant information (i.e., stimulus location in the Simon task). Lower FA rate observed on no-go trials immediately after an incongruent trial can be explained by such a hypothesized inhibition of the response activation at the onset of the no-go stimuli. Provided that the transient modulation of cognitive control is generated primarily through the mechanism of response inhibition, the present findings are consistently explained by the inhibition account.

As mentioned in the introduction, the relationship between the sustained and transient modulations remains controversial. The conflict-monitoring theory proposes that an accumulation of the transient modulation results in the sustained modulation of cognitive control [4,5], whereas other researchers have proposed that these modulations are controlled by different mechanisms such as reactive and proactive control (for the transient and sustained modulations, respectively) [13-15]. In line with the latter argument, Experiments 1 and 2 showed different patterns of response bias in FA, indicating that the sustained modulation is more than a simple accumulation of the transient modulation of cognitive control.

In summary, the present study suggests the dual modulation mechanism of cognitive control in the Simon task. In the transient trial-by-trial sequence, the task-irrelevant information would be inhibited, as expected by the conflict monitoring theory [4,5], the inhibition account [8], and the reactive control [13,14]. As for the sustained conflict context, however, the present results would come to a different conclusion from these accounts. Explanation based on the contingency learning [17,18] would be the best alternative; The task-irrelevant information would be used in the reversed manner based on the contingency learning of association between the stimulus location and the response mapping. These suggest that the modulation of cognitive control is not achieved by the single mechanism. Rather, multiple mechanisms, including the active response bias demonstrated in the present study, should be assumed to work together to enable the adaptive behavior in conflict situations. In the future, to make the present findings to be more general and robust, we need further studies employing wider range of participants with proper sample size considering the effects of their clinical and socio-demographic factors, since the present experiments tested only the university students who are healthy young adults having a certain level of cognitive ability, which is one of the limitations of our study.
